# Optimisation of freeze substitution protocols for the examination of malaria parasite structure by volumetric electron microscopy

**DOI:** 10.1111/jmi.70007

**Published:** 2025-07-09

**Authors:** Rachel Rachid, Camila Wendt, Wanderley de Souza, Kildare Miranda

**Affiliations:** ^1^ Laboratório de Ultraestrutura Celular Hertha Meyer Centro de Pesquisa em Medicina de Precisão Instituto de Biofísica Carlos Chagas Filho and Centro Nacional de Biologia Estrutural e Bioimagem Universidade Federal do Rio de Janeiro Rio de Janeiro Brazil; ^2^ Instituto Nacional de Ciência e Tecnologia em Biologia Estrutural e Bioimagem, Universidade Federal do Rio de Janeiro Rio de Janeiro Brazil; ^3^ Laboratório de Biomineralização Instituto de Ciências Biomédicas Universidade Federal do Rio de Janeiro Rio de Janeiro Brazil; ^4^ Centro Multiusuário para Análise de Fenômenos Biomédicos Universidade do Estado do Amazonas Amazonas Brazil

**Keywords:** freeze substitution, high‐pressure freezing, *Plasmodium*, rodent malaria, transmission electron microscopy

## Abstract

Malaria is one of the deadliest infectious diseases in the world, annually responsible for over 400,000 deaths. It is caused by parasites of the genus *Plasmodium*, which undergo remarkable structural changes during their development within different cells across various hosts. An important approach to understand the structural basis of biochemical and physiological processes during *Plasmodium* infection has been the quantitative measurement of dimensional parameters obtained by different microscopy techniques. In this regard, sample preparation, particularly electron microscopy protocols that rely on room‐temperature chemical fixation, has posed significant challenges, as it is known to produce artefacts such as shrinking, swelling and displacement of structures and osmolytes. In contrast, specimen immobilisation by cryofixation followed by freeze substitution minimises these artefacts and provides better sample preservation. Nevertheless, the composition of the freeze substitution medium may vary depending on the cell type, making it a critical factor for achieving optimal sample preparation. In this work, we optimised a freeze substitution protocol for the structural analysis of intraerythrocytic stages of the murine malaria models *Plasmodium chabaudi* and *P. berghei*. We tested different freeze substitution recipes, considering the biochemical composition of malaria membranes, and compared the results with those obtained through conventional chemical fixation. Overall, the results showed a significant improvement on the preservation of cell morphology and haemozoin crystals. Establishing an efficient and reproducible freeze substitution protocol for murine malaria models provides an important tool for advancing our understanding of the structural organisation of *Plasmodium* spp.

## 1 INTRODUCTION

Malaria is a life‐threatening disease caused by *Plasmodium* spp. Despite continual efforts, the burden of this disease remains substantial. According to the World Health Organization (WHO), 263 million cases and 597,000 deaths were reported in 2021.^1^

The challenges in malaria control are multifaceted. Understanding parasite biology and the structural basis of its interaction with host cells is essential for the development of effective disease control strategies.^2^ In this context, electron microscopy (EM) has proven to be an invaluable tool for studying the fine structure of malaria parasites. The first documented use of EM techniques to the study of *Plasmodium* structure dates back to 1942.^3^ Since then, numerous studies have been published, contributing to our current comprehension of the structure and biology of *Plasmodium* spp.^4^

To better understand biochemical and physiological processes during *Plasmodium* spp. development, quantitative measurements of dimensional parameters across different *Plasmodium* stages have been assessed using various electron microscopy methods.^5^, ^6^, ^7^, ^8^, ^9^, ^10^, ^11^, ^12^, ^13^, ^14^, ^15^, ^16^ For this reason, EM sample preparation protocols able to preserve cell structure are essential.

It has been shown that conventional EM sample preparation methods‐those involving room temperature chemical fixatives such as formaldehyde, glutaraldehyde, and osmium tetroxide, along with dehydration in organic solvents, embedding, sectioning and staining^17^, ^18^ may potentially introduce disturbances that lead to artefacts such as cell or organelle shrinkage, membrane displacement, protein aggregation, and the extraction or mobilisation of small molecules and osmolytes.^19^, ^20^, ^21^ In contrast, cryopreparation methods may help avoid these artefacts. While cryotechniques have their own limitations,^22^, ^23^, ^24^, ^25^ they are considered a gold standard when it comes to preserving cell structure, allowing access to samples in a near‐native state for microscopical analysis.^23^, ^24^ Among the main EM cryotechniques, the analysis of cryofixed samples at a frozen state‐either by conventional electron microscopy of vitrified specimens (CEMOVIS‐two‐dimensional imaging) and cryoelectron tomography (3D imaging) has provided the most impressive data on the fine structure of *Plasmodium* spp.^10^, ^15^, ^26^, ^27^, ^28^, ^29^, ^30^, ^31^, ^32^, ^33^, ^34^, ^35^ In these studies, whole cells adhered to EM grids were plunge‐frozen in liquid ethane and directly observed using CryoTEM, which imposes some limitations regarding cell thickness and sample contrast.^36^, ^37^, ^38^ Recently, a cryolamella preparation workflow for *Plasmodium* spp.‐infected cells using cryo FIB‐SEM has overcome these limitations, providing significantly improved cryotomography datasets.^39^

Although considered the method of choice for ultrastructural characterisation, techniques like cryo FIB‐SEMs and high‐end 200–300 kV instruments require sophisticated, expensive equipment and specific training. In contrast, high‐pressure freezing followed by freeze substitution (HPF‐FS) has proven to be a reliable intermediate method. It combines the superior structural preservation of cryofixation methods with the convenience of room‐temperature 2D and 3D analysis.^10^, ^22^, ^40^, ^41^ During freeze substitution, specimens stabilised by cryofixation are placed in fixatives that are diluted or dissolved in organic solvents kept at low temperatures (i.e., −90°C). This solution permeates the entire sample volume before fixation, and then is gradually warmed up under controlled conditions. The fixatives are then activated, simultaneously fixing structures at different cell levels and depths (from the cortex to the inner nuclear structure), significantly improving structural preservation.^23^, ^42^

Several parameters are known to affect sample preservation. Prior to high‐pressure freezing (HPF), samples should be maintained in buffer and temperature conditions that preserve their viability until cryofixation. Another critical aspect is the use of a cryoprotectant solution to fill the space between the sample and the metal HPF carriers, which is essential to ensure proper vitrification and prevent ice crystal formation.^23^

The choice of substitution medium and the warming up curve are crucial, as the preservation of subcellular structures and membrane contrast can be significantly affected depending on the protocol used.^23^, ^43^ This is particularly true for *Plasmodium* spp. samples, which strictly adhere to the axiom in EM stating that ‘different protocols can result in very different patterns for identical samples’ and present significantly different structure, depending on the sample preparation protocol used.^5^, ^6^, ^7^, ^10^, ^27^

In this work we aimed to optimise a freeze substitution protocol for *Plasmodium* spp. asexual stages, using the murine parasites *P. chabaudi* and *P. berghei* as models. Freeze substitution protocols were tested based on well‐known biochemical composition of certain structures, such as the phospholipid repertoire of their membranes. The results showed a remarkable improvement in ultrastructural preservation. When compared to chemically fixed cells, FS samples appeared almost to correspond to a different cell type. The parasites retained their circular or spherical shape, similar to what is observed on live cell analysis or CEMOVIS imaging. Organelles exhibited a smoother appearance, adjacent membranes were more regularly spaced and a substantial improvement on the preservation of haemozoin crystal morphology was observed.

Overall, the results demonstrate that the use of optimised FS protocols can provide valuable and accurate information about the structure of *Plasmodium* cells, reducing instrument time on high‐end microscopes, a current bottleneck for expanding the low‐temperature structural biology field.

## 2 MATERIAL AND METHODS

### 2.1 Ethics statement

This study was approved by the Ethics Committee for Animal Experimentation of the Health Sciences Centre of the Federal University of Rio de Janeiro, under the Protocol n° 161/19, according to the Brazilian federal law (11.794/2008, Decreto n° 6.899/2009), which is based on the ‘Guide for the Care and Use of Laboratory Animals’ prepared by the National Academy of Sciences, USA, and the ‘Australian Code of Practice for Care and Use of Animal for Scientific Purpose’. All animals received humane care in compliance with the above‐mentioned guides.

### 2.2 Parasites

*Plasmodium chabaudi chabaudi* (AJ) and *P. berghei* (ANKA) were maintained in 6–8 weeks old male CF1 mice. The animals were accommodated in an air‐conditioned environment (22–24°C) in a regular 12‐h light/dark cycle with water and standard feed ad libitum. Parasites were stored in liquid nitrogen between experiments to avoid any selection of virulent strains. After amplification, mice were inoculated by intraperitoneal injection of 10^6^ infected red blood cells (iRBC) with either *P. chabaudi* or *P. berghei*. Peripheral blood parasitaemia was determined by microscopic examination (bright field microscopy) of Giemsa‐stained tail‐blood films. Mice with parasitaemia higher than 20% were used. Animals were euthanised with a combination of ketamine (240 mg/kg body weight) and xylazine (15 mg/kg body weight). Blood was collected via cardiac puncture for analysis.

### 2.3 Room temperature chemical fixation

iRBCs were centrifuged at 1500 × *g* for 5 min and washed twice with 0.1 M PHEM buffer (2.5 mM MgCl_2_, 35 mM KCl, 5 mM EGTA, 10 mM HEPES, 30 mM Pipes, pH 7.2) for 5 min. The cells were fixed in a solution containing 2.5% glutaraldehyde, 4% formaldehyde, 4% sucrose in 0.1 M PHEM buffer and stored overnight at −4° C. After fixation, samples were washed twice in 0.1 M PHEM buffer and postfixed in 1% OsO_4_ plus 0.8% ferrocyanide and 5 mM CaCl_2_ in 0.1 M cacodylate buffer for 40 min. Dehydration was performed using a graded ethanol (CAS #64‐17‐5, Merck, Darmstadt, Germany) series (30%, 50%, 70%, 90%, and 100%) for 10 min at each step. Subsequently, samples were infiltrated and embedded in EMbed 812 epoxy resin (CAS #14120, Electron Microscopy Sciences—EMS, Pennsylvania, USA). The resin was prepared according to the data sheet to achieve medium hardness. Infiltration was carried out in a graded ethanol‐to‐resin ratio of 2:1, 1:1, and 1:2, with each step lasting 12 h at room temperature. At the end the samples were placed in pure resin, transferred to a flat embedding mould, and polymerised at 60°C for 48 h.

### 2.4 High‐pressure freezing

iRBCs were centrifuged at 1500 × *g* for 5 min and inserted by capillarity in 2 mm pieces of 200 µm diameter cellulose capillaries (Bal‐Tec, Co., Liechtenstein). One end of the capillary was closed using tweezers. Four capillaries were mounted between the two aluminium carriers (3×0.5 mm) (Bal‐Tec, Co., Liechtenstein). The pellet was placed between two types of carriers (type A and B) so that the cells were protected in a 200 µm cavity on one carrier. The cavities were filled with 1‐hexadecane to avoid air between the capillaries. The sandwiched samples were mounted in the high pressure freezing (HPF) holder and frozen using a Bal‐Tec HPM 010 HPF machine (Bal‐Tec, Co., Liechtenstein). After freezing, the samples were stored in liquid nitrogen.

### 2.5 Freeze substitution and embedding

Samples were carefully removed from liquid nitrogen and immersed in the freeze substitution (FS) medium. Five different FS media were tested, with their specific compositions detailed in Table 1. Absolute acetone (CAS #67‐64‐1, Merck, Darmstadt, Germany) was used as the diluent in all tested media. Osmium tetroxide (CAS #20816‐12‐0, Electron Microscopy Sciences—EMS, Pennsylvania, USA) was diluted in absolute acetone to prepare 10 mL of a 4% stock solution, aliquoted into cryotubes, and stored in liquid nitrogen. Similarly, uranyl acetate (CAS #541‐09‐3, EMS, Pennsylvania, USA) was diluted in absolute acetone to a 1% stock solution and stored at −20°C. Aqueous glutaraldehyde (Grade 70%, CAS #111‐30‐8, EMS, Pennsylvania, USA) was also stored at −20°C. FS media were prepared from these stock solutions, aliquoted into cryotubes, and stored in liquid nitrogen. Fresh Milli‐Q water was used in media containing water.

**TABLE 1:** Composition of FS mediums. Acetone absolute was used as diluent. Even though water was not added to the FS medium 3, it is worth to note that there is still a trace amount of water derived from the 70% aqueous glutaraldehyde stock solution.

FS medium	Osmium	Glutaraldehyde	Uranyl acetate	Water
1	1%	X	0.5%	1%
2	1%	X	0.5%	3%
3	1%	0.5%	X	X
4	1%	0.5%	0.5%	1%
5	1%	1%	0.5%	1%

Freeze substitution was performed using a Leica AFS2 system (Leica Microsystems, Vienna, Austria). The chamber was pre‐cooled to −90°C and filled with absolute ethanol (CAS #64‐17‐5, Merck, Darmstadt, Germany) to enhance heat exchange. Cryotubes containing 1 mL of FS medium were transferred to the Leica AFS2 chamber and kept at −90°C for 30 min. After this period, the FS medium was in the liquid state and the chamber temperature was stabilised. The samples were transferred to the system. The aluminium carriers containing the frozen capillaries were carefully placed in the different FS media using pre‐cooled tweezers, and the FS program was initiated.

The warming up curve (Graphic 1) consisted of maintaining the samples at −80°C for 72 h, followed by a gradual warming to −20°C over 20 h (curve slope 3). The samples were then held at −20°C for 4 h before being further warmed to 4°C over 4 h (curve slope 6). Finally, the samples were maintained at 4°C for 4 h.

**GRAPHIC 1:**
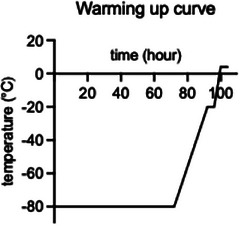
Warming‐Up Curve for Freeze Substitution. Samples were kept at −80°C for 72 h before being gradually warmed to −20°C over 20 h and held at this temperature for 4 h. Subsequently, the temperature was increased to 4°C over 4 h and then maintained at 4°C for an additional 4 h.

Following freeze substitution, samples were washed three times with absolute acetone (CAS #67‐64‐1, Merck, Darmstadt, Germany) at room temperature for 5 min each and then stepwise embedded in EMbed 812 epoxy resin (CAS #14120, EMS, Pennsylvania, USA). The resin was prepared according to the data sheet to achieve medium hardness. Infiltration was carried out in a graded ethanol‐to‐resin ratio of 2:1, 1:1, and 1:2, with each step lasting 12 h at room temperature. At the end the samples were placed in pure resin, transferred to a flat embedding mould, and polymerised at 60°C for 48 h.

For this study, samples were processed in three independent experiments for each condition. In each experiment, one infected mouse was used. The blood obtained from the infected mouse was split into two portions: one was subjected to the chemical fixation protocol, while the other was used for HPF‐FS experiments. Each HPF carrier contained two capillaries, and each FS medium was tested in capillaries from two different carriers. The efficiency of the HPF process was evaluated by analysing the temperature and pressure curves, which was displayed on an oscilloscope connected to the HPF equipment. We observed that the equipment efficiency decreases after 15 individual freezing cycles. To ensure reproducible results, we established a maximum of 12 freezing cycles per experiment. Both chemically fixed and HPF‐FS samples were infiltrated and polymerised using the same resin formulation. At least three samples from each condition were imaged.

### 2.6 Transmission electron microscopy (TEM)

Thin sections (70 nm) were obtained and stained for 20 min in 5% aqueous uranyl acetate and for 5 min in lead citrate and observed in a Tecnai Spirit (Thermo Fisher Scientific, USA) electron microscope operating at 120 kV equipped with a Veleta 2K camera (Olympus).

Morphometric analysis was carried out using ImageJ software (US National Institutes of Health, Bethesda, Maryland, USA). The circularity index (*Ci*) was calculated using the equation: *Ci* = 4*π* × (*Acircle*) / (*Pcircle* ^2^). The analysis was conducted on samples prepared using the chemical fixation protocol and to samples processed by HPF followed by freeze substitution in the optimised medium (FS medium 5: 1% OsO₄, 1% glutaraldehyde, 0.5% uranyl acetate, and 1% water). An average of 40 randomly selected profiles of iRBCs and *P. chabaudi* parasites were manually segmented and quantified using ImageJ measurement tool.

Statistical analyses were carried out using Prism 9 (GraphPad Software Inc., La Jolla, CA, USA). Normality analysis with Shapiro‐Wilk test indicated that the measured data did not support the assumption of a Gaussian distribution of shape‐based measures. Samples were further analysed using nonparametric statistical tests followed by Mann–Whitney test.

### 2.7 Transmission electron tomography

Two‐hundred nanometres sections were cut and collected as serial sections on Formvar‐coated slot copper grids. Samples were post‐stained for 20 min in 5% aqueous uranyl acetate, for 5 min in lead citrate and incubated with 10 nm colloidal gold on both sides for 5 min and washed in distilled water. Sections were observed in a 200 kV FEI Tecnai G2 transmission electron microscope (Tecnai G2, Thermofisher) equipped with a 4 k CEMOS camera (AMT‐USA). Tilt series were acquired using Xplore 3D (FEI Company, Thermofisher) or SerialEM.^45^ Tomograms were recorded between −65° and + 65° with an angular increment of 1°. Alignments were applied using fiducial markers and weighted back projections with the IMOD software package.^45^, ^46^ The IMOD package was used for segmentation and data display.

### 2.8 FIB‐SEM

Resin embedded samples were mounted on a stub, coated with a 10 nm gold layer and observed in an Auriga cross beam scanning electron microscope (Zeiss, Oberkochen, Germany). A U‐shaped trench was cut around the area of interest using an ion beam operating at 30 kV and a current of 16 nA for coarse milling. The exposed surface was polished with a lower ion bean current (2 nA) and images were sequentially collected in ‘slice‐and‐view’ mode at an accelerating voltage of 1.8 kV and a beam current of 0.8 nA. Image acquisition was done in the immersion lens mode, using a CBS (Concentric Back Scatter) detector. Image stacks were acquired with a dwell time of 4 µs and using the line average (value of 40). Image store resolution was of 3072×2304 pixels with a voxel size of 15×15×30 nm. FIB‐SEM series were aligned using the IMOD software package.^45^, ^46^ Image segmentation and 3D rendering was done with Amira software (Thermo Fisher Scientific, USA).

## 3 RESULTS

**FIGURE 1:**
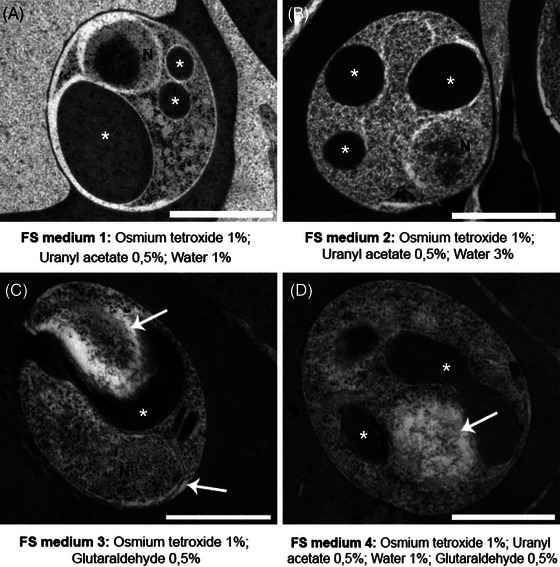
Comparison of *P. chabaudi* high‐pressure frozen parasites freeze‐substituted with different medium compositions. Cellular features as the nucleus (N) and haemoglobin containing structures (asterisk) are discernable. Membranes appear with inverted contrast in samples prepared without glutaraldehyde (A, B), while samples without water (C, arrow) show reduced preservation of internal structures. FS medium 4 (D) shows improved overall preservation compared to other tested media, though some intracellular structures remain poorly defined (D, arrow). N: nucleus, *Haemoglobin containing structures. Scale bar: 1 µm.

Five different freeze substitution (FS) media were tested on high pressure frozen erythrocytes infected with *P. chabaudi*. All tested media used acetone as the diluent, with varying concentrations of osmium tetroxide, glutaraldehyde, uranyl acetate and water (Figure 1). Overall, the HPF‐FS parasites exhibited a round‐shaped morphology. However, differences in membrane contrast and the preservation of internal structures were observed. The use of freeze substitution with a medium composed of osmium tetroxide, uranyl acetate and water resulted in cells with an inverted membrane contrast (Figure 1A and B). FS medium containing glutaraldehyde and osmium tetroxide presented contrasted membranes (Figure 1C, arrow); however, cellular internal structures were poorly preserved. FS medium 4, which was a combination of previous tested media consisted of glutaraldehyde, osmium tetroxide, uranyl acetate and water (Figure 1D); presented the best results among the four tested media, although some intracellular structures were still not well preserved (Figure 1D, arrow).

**FIGURE 2:**
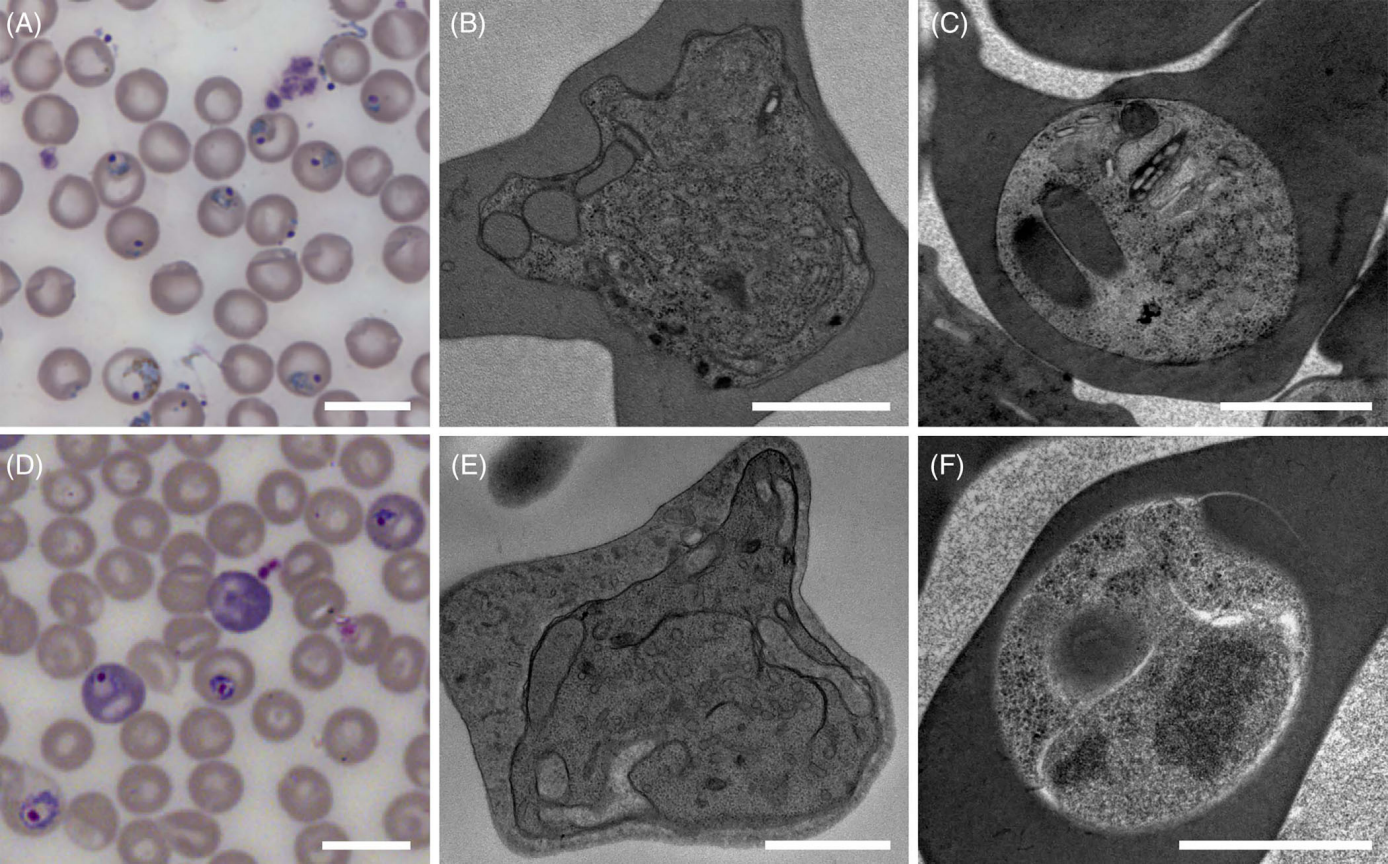
General comparison of *P. chabaudi* (A–C) and *P. berghei* (D–F) trophozoites prepared using conventional electron microscopy protocols and HPF‐FS methods. Trophozoites observed in Giemsa‐stained blood smears (A, D). TEM analysis of chemically fixed (B, E) and HPF‐FS parasites (C, F). Scale bars: 10 µm (A, D); 500 nm (B, C, E, F).

Transmission electron microscopy (TEM) observations of the four initially tested media showed the medium 4 as the most promising. Previous works on the biochemical composition of *P. chabaudi* membranes have revealed that parasite plasma membrane is primarily composed of phosphatidylcholine and phosphatidylethanolamine.^47^ Based on the analysis by Wunderlich *et al.*, we increased the glutaraldehyde concentration on the FS medium 5, as it is known to react with the amino groups of proteins and the amino radicals of phospholipids. This increase of glutaraldehyde concentration resulted in cells with a well‐preserved ultrastructure (Figure 2). Nevertheless, artefacts due to membrane shrinkage were still observed, resulting on electron lucent spaces close to the nuclear membrane (Figure 2F). Given the positive results obtained in preserving parasite ultrastructure, all subsequent analyses in this work were based on the comparison between chemically fixed parasites (referred to as the ‘conventional electron microscopy protocol’) and HPF‐FS parasites processed with freeze substitution medium 5

**FIGURE 3:**
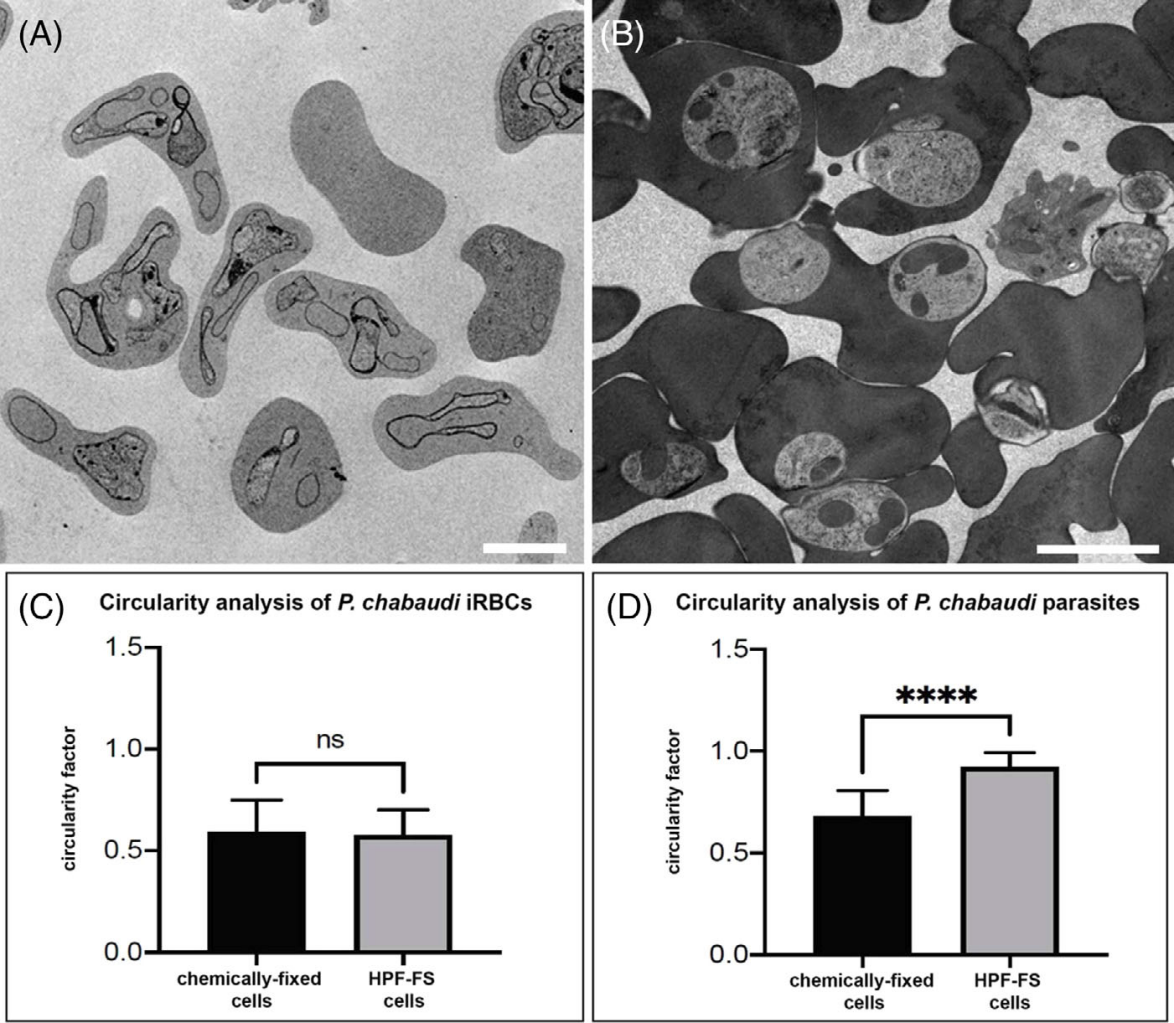
Lower‐magnification TEM images of *P. chabaudi* samples prepared using chemical fixation (CF) (A) and HPF‐FS with freeze substitution medium 5 (B). Circularity analysis was performed on an average of 40 cells for each condition. Scale bar: 1 µm.

The general morphology of high‐pressure frozen and freeze substituted samples was assessed by TEM (Figure 2). Prior to HPF and freeze substitution, cells were observed by light microscopy. Analysis of Giemsa‐stained blood smears from *P. chabaudi* (Figure 2A) and P *. berghei* (Figure 2D) infected animals showed round shaped cells occupying most of the erythrocyte area. At this magnification, the nucleus appears as a circular dark purple structure, whereas the parasite cytoplasm is visible in light purple. This is a standard method in malaria research and diagnosis and was included here for comparison purposes. Transmission electron microscopy observation of cells prepared following room temperature chemical fixation protocols, referred here as conventional electron microscopy methods (Figure 2B and E), revealed cells with an amoeboid shape, surface distortions in both the cell cortex and intracellular structures, and content extraction typical of malaria parasite preparations for TEM. These distortions and extraction varied in severity but were present in all observed cells, resulting in a morphological organisation substantially different from that seen in freshly prepared blood smears. In contrast, HPF‐FS cells exhibited a circular shape (Figure 2C and F), closely resembling the morphology commonly seen on blood smears. Differences in host cell contrast were also evident (Figures 2 and 3A and B). The differences were observed comparing parasites at the trophozoite stage. Chemically fixed erythrocytes displayed a lighter cytosolic contrast, whereas cryofixed samples showed a denser cytoplasm, likely reflecting improved preservation of haemoglobin content.

**FIGURE 4:**
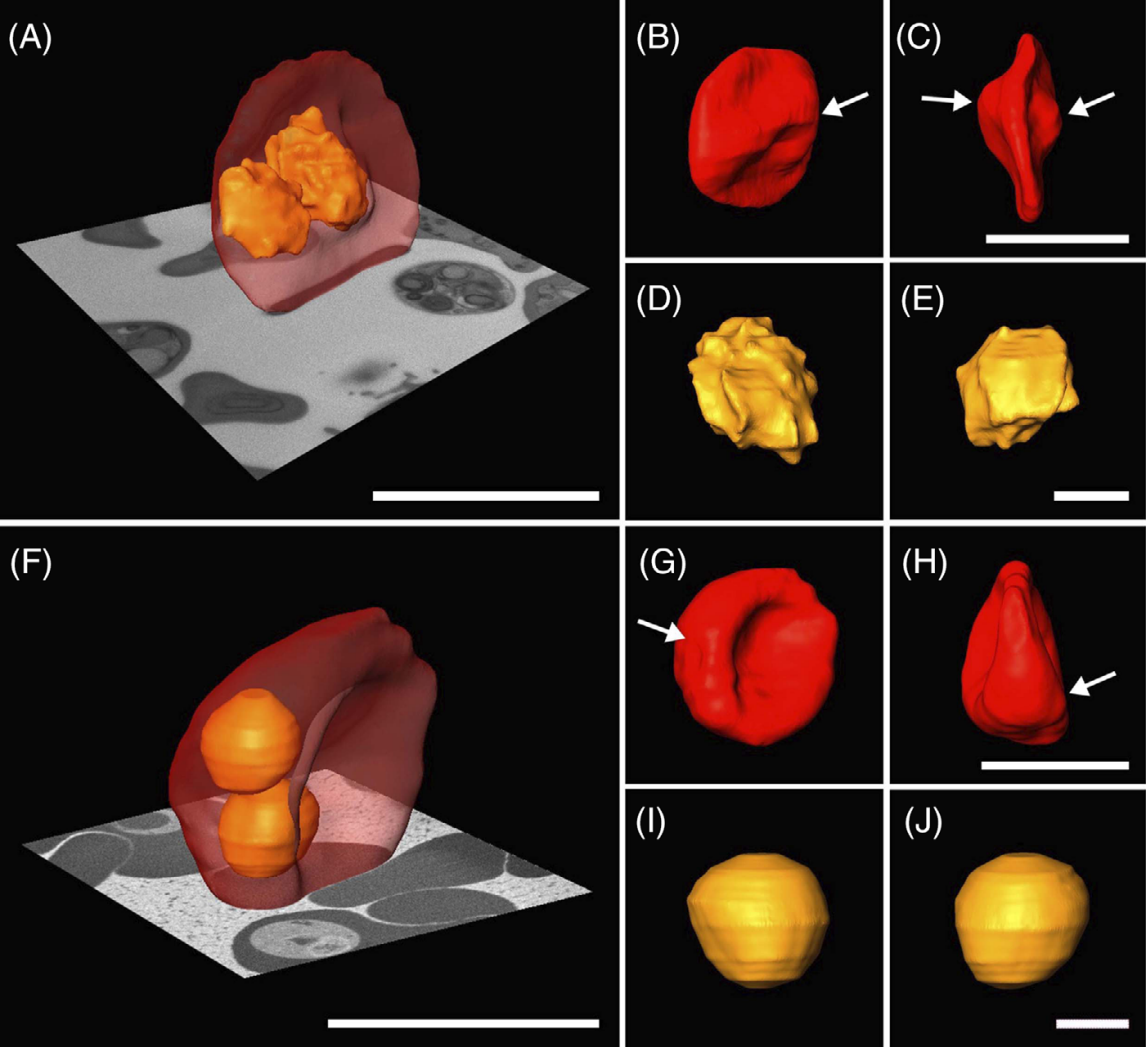
FIB‐SEM 3D reconstruction of erythrocytes infected with *P. chabaudi* trophozoite stages submitted to CF (A–E) or HPF‐FS (F–J) protocols. Representative images illustrate differences in cell shape between CF (D, E) and HPF‐FS (I, J) samples. Arrows indicate deformations in the host cell shape caused by the presence of the parasite. Scale Bar: 5 µm (A–C, F–H); 1 µm (D, E, I, J).

The general morphology of chemically fixed (CF) and HPF‐FS samples was further analysed using two‐dimensional TEM images (Figure 3) and 3D‐rendered models generated by FIB‐SEM reconstructions (Figure 4). Differences in cell morphology were assessed using ImageJ circularity analysis (Figure 3C and D). A circularity value of 1 indicates a perfect circle, while values closer to 0 represent increasingly elongated shapes. No significant differences were observed between CF and HPF‐FS infected erythrocytes (Figure 3C). However, a significant difference was found between CF and HPF‐FS *P. chabaudi* parasites (Figure 3D), supporting the observations from TEM images (Figure 3A and B).

**FIGURE 5:**
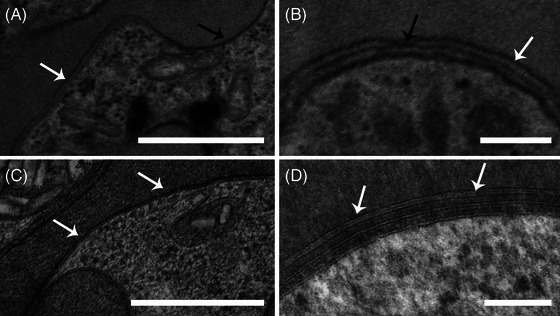
Comparison of membrane bilayer preservation in *P. chabaudi* trophozoite (A–C) and schizont (B–D) stages. CF samples (A, B) showed varying degrees of lipid bilayer preservation, with PVM and PPM often poorly discernible or unresolved. In contrast, HPF‐FS samples (C, D) presented smoother and well discernable membranes. Scale bar: 500 nm (A, C); 100 nm (B, D).

**FIGURE 6:**
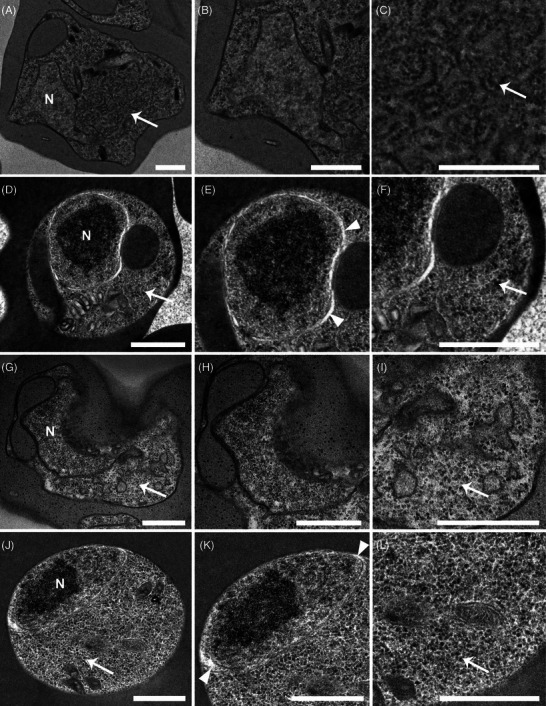
Comparison of nuclei, ribosomes and endoplasmic reticulum (ER) structures in chemically fixed (CF) and high‐pressure frozen, freeze‐substituted (HPF‐FS) cells. *P. chabaudi* (A–F) and *P. berghei* (G–L) samples were analysed. In CF cells (B, H), the nuclei exhibited poor structural definition, while HPF‐FS samples (E, K) displayed euchromatin and heterochromatin regions. ER membranes in CF cells (C, I; arrows) were more evident due to the extraction of lumen content during processing, which enhanced membrane contrast. In HPF‐FS cells (F, L; arrows), the ribosomes appeared denser, with no discernible ER membranes. A visible separation of the nuclear envelope membranes is observed in HPF‐FS (E, K; arrowheads). N: nucleus. Scale bars: 500 nm.

**FIGURE 7:**
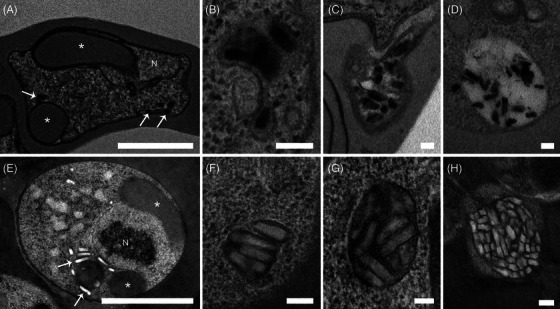
Haemozoin crystal morphology in *P. chabaudi* submitted to chemical fixation (A–D, arrows) and HPF‐FS (E–H, arrows). Haemozoin crystals are seen as electron dense structures in conventional prepared cells whereas in HPF‐FS cells they are electron transparent. N: nucleus, *: haemoglobin containing structures. Scale bars: 1 µm (A, E); 100 nm (B–D, F–H).

**FIGURE 8:**
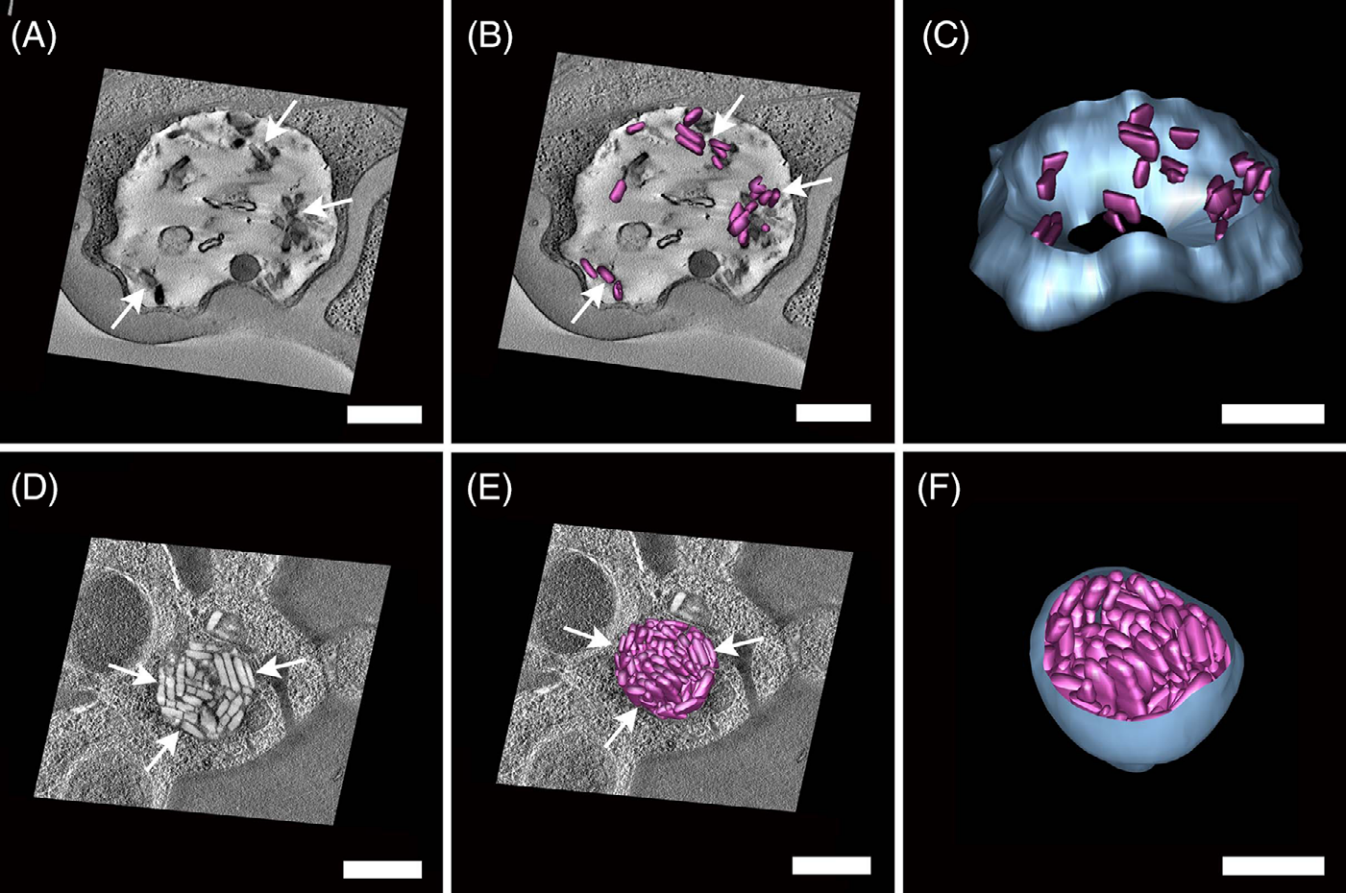
Preservation of food vacuole internal content in *P. chabaudi* submitted to CF (A–C) and HPF‐FS (D–F). Differences in food vacuole shape and internal content were evaluated by serial section electron tomography. CF cells presented a food vacuole with ruffled membrane (A–C) and fewer haemozoin crystals inside (A and B, arrows; C). In contrast, HPF‐FS cells have food vacuoles with smoother membrane (D–F) and completely filled with haemozoin crystals (D–F, arrows; F). Light blue: food vacuole, purple: haemozoin crystals. Scale bar: 500 nm.

Analysis of FIB‐SEM 3D rendered models showed the polymorphic shape of erythrocytes in both CF and HPF‐FS samples (Figure 4A–C, F–H). Deformations on the surface of red blood cells due to the presence of the parasites were clearly visible (Figure 4B and C and G and H; arrows). Parasite structure reproduces the results previous observed by 2D analysis (Figure 4I and J). The improvement seen on HPF‐FS cells is noticed on the cell shape, which is round, resembling what is seen on Giemsa‐stained blood smears. CF parasites (Figure 4D and E) generally exhibited body shrinking, as also observed in transmission electron microscopy analysis (Figures 2 and 3). FIB‐SEM image stacks were acquired at lower magnification, as to provide an overview of multiple infected erythrocytes (Figure S1). This approach impacted the resolution of 2D slices and the visualisation of organelles. Nevertheless, fine ultrastructural details were explored by TEM (Figures 5, 6, 7) and electron tomography (Figure 8).

Membrane structure was notably better preserved in HPF‐FS cells. This is critical for structural analysis of *Plasmodium* spp., as intraerythrocytic stages reside within a parasitophorous vacuole formed during invasion. The parasitophorous vacuole membrane (PVM) is usually closely apposed to the parasite plasma membrane (PPM) in ring and trophozoite stages. At the schizont stage, a third membrane emerges, corresponding to the newly formed merozoite plasma membrane. TEM analysis of CF and HPF‐FS *P. chabaudi* trophozoites (Figure 5A) and schizonts (Figure 5B) showed different degrees of membrane preservation. In CF cells, the PVM and PPM frequently lacked bilayer resolution or were poorly distinguishable (Figure 5A and B, white arrows), particularly in regions where sectioning plane was unfavourable for their separation as distinct entities (Figure 5A and B, black arrows). In contrast, cryofixed samples (Figure 5C and D), consistently showed well‐resolved bilayer structures for both the PVM and PPM, providing smoother, clearly defined, and discernable membranous structures.

**FIGURE 9:**
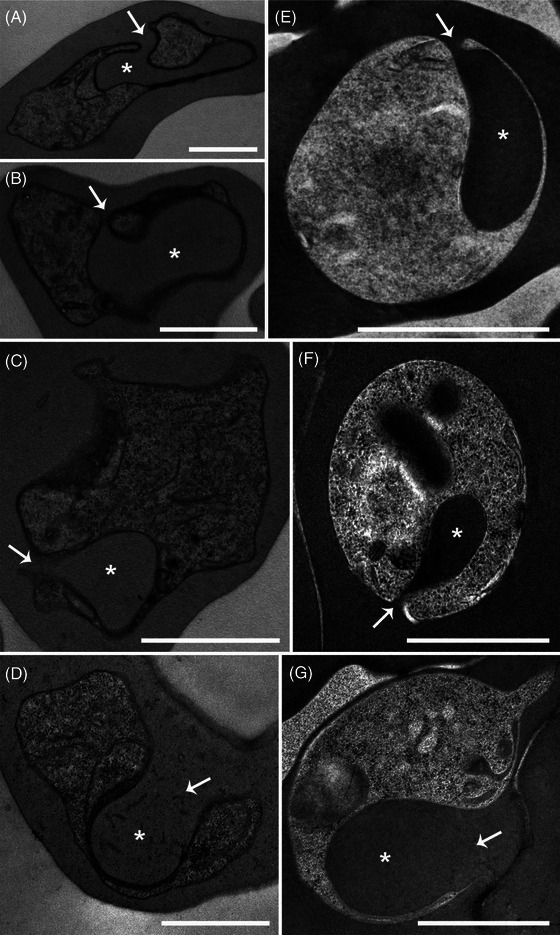
Haemoglobin uptake mechanisms of *P. chabaudi* (A–C, E, F) and *P. berghei* (D, G) trophozoites fixed by conventional (CF) methods and HPF‐FS. A large cytostome tube (asterisk), which extends through the cytoplasm, originates from the cytostome aperture (arrows). Differences in cytostome tube morphology are observed between samples prepared using conventional electron microscopy protocols (A–D) and those processed by cryofixation followed by freeze substitution (E–G). Scale bar: 500 nm.

Overall, subcellular structures were better preserved in HPF‐FS samples from different *Plasmodium* species (Figure 6). In *P. chabaudi* and *P. berghei* cells, nuclei appeared as turgid, rounded structures with clear distinction between euchromatin and the heterochromatin regions (Figure 6D and E and J and K). This was clearly not the case in CF cells (Figure 6A and B and G and H), where the nuclear structure was poorly defined and difficult to visualise. Nevertheless, separation of the nuclear envelope lumen is observed on HPF‐FS samples (E, K; arrowheads). This effect is likely due to uneven expansion during resin infiltration steps or osmotic effect just before freezing. Interestingly, the endoplasmic reticulum (ER) was more easily visualised in CF cells (Figure 6C), likely due to the extraction of the ER matrix content during dehydration, which enhanced the contrast between the ER membrane (and adhered ribosomes) and its lumen. Conversely, HPF‐FS cells, showed a dense number of ribosomes (Figure 6F and L), with no distinguishable ER membrane. Additionally, pepper precipitation artefacts were observed on CF cells (Figures 6G and H and 9D). The small, particulated material observed within the host cell cytoplasm is related to a chemical reaction that occurred between osmium and host cell haemoglobin, resulting in osmium precipitation.

Remarkable differences were also observed in haemozoin crystals (Figure 7). As crystalline structures, haemozoin crystals may lose part of their content during the dehydration steps in conventional electron microscopy sample preparation. The overall crystal shape, seen as small rectangular shaped electron‐dense structures, did not change across the different fixation methodologies (Figure 7A–D, arrows). Nevertheless, crystal edges were better preserved in HPF‐FS cells although less contrasted (electron transparent) when compared to RT chemically fixed crystals (Figure 7E–H, arrows).

Differences in haemozoin crystal preservation were further assessed by serial section TEM tomography (Figures 8 and S2). CF *P. chabaudi* exhibited a ruffle membrane with few haemozoin crystals inside (Figure 8A–C, arrows), whereas HPF‐FS parasites had food vacuoles with smoother membranes, and their internal content was completely filled with haemozoin crystals (Figure 8D–F, arrows). The contrast of haemozoin crystals inside the food vacuole between CF and HPF‐FS samples is drastic in 3D rendered models (Figure 8C and F).

Structures involved in haemoglobin uptake in *Plasmodium* spp. trophozoites are also affected by CF (Figure 9). Haemoglobin uptake in *Plasmodium* spp. trophozoites occurs via specialised structures, known as cytostomes. These are formed after invagination of the PVM and PPM, originating a haemoglobin‐filled tube with two apposed membranes that deeply project into the parasite body. Subsequently, a single membrane bound food vacuole is formed, where haemoglobin is digested and, by an unknown process, one of the bilayers (PVM or PPM) is lost (removed or digested). Understanding of such a complex process is highly dependent on the preservation of cell structure, and the use of HPF‐FS protocols has tremendous potential to contribute with preliminary data that may be confirmed by other high‐end techniques such as cryotomography. Haemoglobin uptake mechanisms both in *P. chabaudi* (Figure 9A–C and E and F) and *P. berghei* (Figure 9D and G) trophozoites were dramatically affected by CF, resulting in deformed cytostomal tubes (Figure 9A–D, asterisks) with various shapes, swirls and orientations. In contrast, cells submitted to the HPF‐FS protocol showed well‐preserved cytostomes, with a smooth tubular structure, always projecting a single (in some cases two) arm oriented towards the cell body (Figure 9B and D; arrows). As bilayer resolution is easily achieved with such samples, this approach might represent a step forward in strengthening the repertoire of tools for morphofunctional characterisation of the origin of the food vacuole membrane.

**FIGURE 10:**
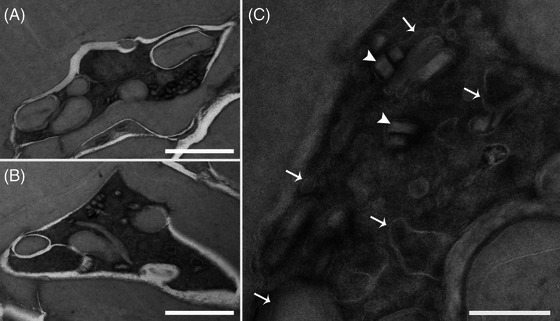
Changes in specific time steps of the warming up curve affect cell shape and membrane contrast. *P. chabaudi* trophozoites were high pressure frozen and freeze substituted using a modified warming up curve. Reducing the time at the −20°C to 4°C step to half of the originally established duration caused alterations in cell morphology (A, B). Although haemozoin crystals remained electron‐lucent (C, arrowheads), cell membranes exhibited an inverted contrast (C, arrows). Scale bar: 500 nm.

In order to optimise the time necessary for an efficient freeze substitution process, changes were made in the warming curve during infiltration and fixation steps. Reducing the time from 72 to 48 h in the first step (−80° to −20°C) did not significantly alter cell morphology or its subcellular structure (data not shown). However, reducing the time from 24 to 12 h at temperatures between −20° and 4°C directly affected cell shape, with amoeboid‐shaped cells frequently observed (Figure 10). These resembled cells seen in preparations using conventional electron microscopy following room temperature protocols. Despite the altered cell shape, membranes and subcellular structures, such as the haemozoin crystals, exhibited structural preservation similar to those seen in cells that were kept for 24 h at the −20° to 4°C step. In addition, electron‐lucent membranes were seen (Figure 10C, arrows), in contrast to those observed in preparations following the original protocol.

## 4 DISCUSSION

In this work, we used cryofixation by high‐pressure freezing followed by freeze substitution to improve the ultrastructural preservation of *P. chabaudi* and *P. berghei* asexual stages. Although different FS protocols have been successfully applied to *Plasmodium* spp. cryofixed samples (Table 2),^7^, ^10^, ^27^, ^32^, ^48^, ^49^, ^50^, ^51^, ^52^, ^53^, ^54^ optimisation of protocols based on the membrane composition have been so far not documented in the literature. A detailed description of freeze substitution methodology, including the FS medium composition and the warming curve, is essential for ensuring reproducibility of this technique.

**TABLE 2:** List of freeze substitution media and embedding resin used on *Plasmodium* spp.

FS‐medium	Warm up curve	Embedding	Reference
acetone	Non specified	Lowicryl	Waller et al., 2000
0.1% glutaraldehyde, 0.25% uranyl acetate and 0.01% osmium tetroxide in acetone	Gradual temperature increase of 10°C/h	Lowicryl	Henrich et al., 2009
2% glutaraldehyde and 0.1% tannic in acetone followed by incubation with 0.1% uranyl acetate and osmium tetroxide 1% for 1 h on ice after finishing warm up curve	Gradual temperature increase of −90°C to −30°C over 90 h	Epoxy	Weiner et al., 2011 Kapishnikov et al., 2012
1% uranyl acetate in acetone	−90°C for 24 h followed by resin embedding and polymerisation at −45°C for 48 h	Lowicryl	Lemgruber et al., 2013 Hansen et al., 2013
0.2% uranyl acetate	Non specified	Lowicryl	Watermeyer et al., 2016 Hale et al., 2017
0.3% uranyl acetate in acetone	24 h at −90°C, followed by an increase in temperature from −90°C to −45°C in 9 h (5°C/h). Resin embedding and polymerisation at −45°C	Lowicryl	Simon et al., 2021
Lowicryl HM20 resin with 0.2% uranyl acetate	Non specified	Lowicryl	Perrin et al., 2021

The freeze substitution media tested in this work were selected based on protocols from the literature (Table 2) and the biochemical composition *Plasmodium* spp. membranes. Malaria‐infected erythrocytes exhibit significant alterations in membrane composition, including higher levels of phosphatidylcholine and phosphatidylinositol, and lower levels of sphingomyelin compared to uninfected erythrocytes. Similarly, malaria parasites themselves also contain less sphingomyelin and more phosphatidylcholine and phosphatidylethanolamine.^47^, ^55^ The optimised FS medium used in this work was composed of 1% osmium tetroxide, 1% glutaraldehyde, 0.5% uranyl acetate and 1% of water in acetone. Glutaraldehyde is a fixative that reacts with amino groups of proteins and amino radicals of phospholipids such as phosphatyldyserine and phosphathidylethanolamine.^56^, ^57^, ^58^ Its inclusion in the FS medium is important for stabilising the parasite plasma membrane. In contrast, osmium tetroxide promotes oxidation of unsaturated lipid bonds, such as those in sphingomyelin present in the host cell plasma membrane. In this sense, glutaraldehyde and osmium stabilisation at low temperatures helps preserve of erythrocyte internal content, resulting in the electron dense host cell cytosol that was observed on HPF‐FS samples.

Another key point of discussion is the freeze substitution warming‐up curve. The rationale for extended FS was originally proposed to prevent recrystallisation during the warm‐up phase.^23^, ^24^ Model calculations indicated that the inclusion of water in the FS medium delayed substitution by several hours.^43^ However, recent works have shown that substitution can be much faster while still yielding well‐preserved samples.^24^, ^42^, ^59^ Attempts to shorten the FS curve during the −20° to 4°C step drastically altered cell morphology. At −20°C uranyl acetate remains active, stabilising lipids against extraction, which may also be beneficial for *Plasmodium* spp.^24^

The advantages and limitations of high‐pressure freezing (HPF) and freeze substitution (FS) have been extensively discussed in the literature.^23^, ^24^, ^42^, ^43^, ^59^ Most ultrastructural studies of *Plasmodium* spp. using HPF‐FS focused more on addressing specific biological questions than on the sample preparation methods themselves. Significant insights have been gained, particularly by examining the merozoite^10^, ^27^ and schizont^51^, ^53^, ^54^ stages. Investigations at the trophozoite stage typically concentrated on specific structures such as Maurer's clefts,^32^, ^52^ the nuclear pore,^49^ the apicoplast,^27^, ^49^ and haemozoin crystals.^51^

Overall, there is a consensus that HPF‐FS protocols minimise structural distortions and reduce the extraction of cellular components. Hanssen et al.^10^ specifically compared merozoite invasion of host cells using different fixation strategies, including chemical fixation, HPF‐FS and cryo‐electron microscopy (cryo‐EM). A major advantage of employing multiple fixation methods is the ability to distinguish artefacts that are unique to each approach.

Taking our data with that of Hanssen et al.,^10^ it appears that cellular morphology observed in HPF‐FS and cryo‐EM samples is largely comparable. Nevertheless, the authors mention that protein segregation between the rhoptry neck and bulb, which are visible in chemically fixed and HPF‐FS samples are not detected in cryo‐EM data. With recent advances in cryo‐electron microscopy instrumentation, invasion‐related organelles present on the merozoite stage have been revisited using sub‐tomogram averaging approaches, yielding new insights into the molecular organisation of the invasion apparatus.^34^, ^35^

Analysis of previously published HPF‐FS datasets on malaria parasites revealed cells exhibiting inverted membrane contrast.^51^, ^52^, ^53^, ^54^ A notable difference between those protocols and the one employed in the present study is the absence of water in the substitution medium. Previous studies have shown that the visibility of the biological membranes generally improves when the substitution medium contains 1%–5% water.^43^ However, the precise mechanism by which water affects membrane visibility remains unclear. In this work, we observed that a FS medium consisting solely of osmium tetroxide and water resulted in membranes with inverted contrast, whereas this issue was not observed in the FS medium containing only glutaraldehyde and osmium tetroxide. Membrane contrast variability in osmium‐based FS protocols is not unprecedented. Previous studies^60^, ^61^ documented cases in which membranes appear pale against a more electron‐dense background, even when classical osmium–acetone protocols are applied.

Results similar to those obtained in the present study have been reported in protocols employing uranyl acetate followed by Lowicryl resin embedding. The most notable similarities were in membrane contrast and ultrastructure preservation. Indeed, uranyl acetate is frequently used alone in acetone or methanol in low‐temperature embedding protocols.^42^, ^62^ This strategy has proven effective for improving membrane visualisation, resin infiltration, and also for preserving antigenicity, making it suitable for subsequent immunolabelling applications.^61^ However, embedding at low temperatures presents technical challenges, particularly in preventing condensation of water on the resin and moulds.^24^ Although we did not test embedding in methacrylate‐based resins at low temperatures in this study, comparable results were achieved.

A notable difference was observed in the preservation of haemozoin crystals. This structure has been specifically investigated in the work of Kapishnikov et al.,^51^ although haemozoin crystals are also visible in low‐magnification images reported in other studies.^49^, ^54^ Interestingly, haemozoin crystals with an electron‐lucent morphology have only been observed in our work and that of Kapishnikov et al.

Given the crystalline nature of haemozoin, significant alteration by HPF‐FS protocols was not anticipated. Nonetheless, we observed differences in both the number and morphology of crystals within the food vacuole. Conventional electron microscopy involves a dehydration process at room temperature using organic solvents, which often lead to the partial loss of the ionic content within cells.^23^ Consequently, haemozoin crystals are poorly preserved. In chemically fixed samples, food vacuoles appeared only partially filled with haemozoin crystals. In contrast, HPF‐FS cells at the same development phase (late trophozoites and schizonts) exhibited food vacuoles filled with haemozoin crystals. In addition, haemozoin crystals edges were clearly visible in cryofixed cells, allowing a better analysis of the crystal shape as well as the determination of crystal growing areas. The FS protocol used in this study resulted in food vacuoles with haemozoin dispersion and morphology that resemble those obtained by cryo‐electron microscopy,^63^, ^64^ in contrast to those reported using conventional chemical fixation.^65^, ^66^

It is important to note, however, that due to its crystalline structure, one would not expect that it would completely disappear during sample preparation. That said, when dealing with biological crystals, these observations warrant further discussion. Biological crystals are known to be more sensitive to dehydration agents, and this vulnerability has driven the increasing adoption of cryofixation techniques in studies aiming to avoid artefacts and preserve the integrity of mineral structures.^67^, ^68^, ^69^

Accurate interpretation of *Plasmodium* spp. fine structure is crucial for understanding may aspects of the cell biology of the parasite. Recently, haemoglobin uptake mechanisms in *P. falciparum* trophozoite stages have been revisited,^13^, ^14^, ^16^, ^70^, ^71^, resulting in conflicting observations. In addition to the classical cytostome‐mediated haemoglobin uptake mechanism initially described in malaria parasites,^65^ large haemoglobin‐containing structures, with a phagocytosis‐like uptake have also been observed.^14^, ^16^ Notably, the absence of a microtubule support structure in cytostomal tubes renders them less stable.^65^ In this regard, it is possible that tube morphology may also be more affected by membrane shrinkage artefacts. Such dramatic changes in cytostomal tube morphology could lead to misinterpretation of the steps involved in haemoglobin uptake in these organisms. Proper sample preservation is essential for accurately evaluating the biological effect of antimalarial drugs, particularly those targeting the biogenesis of haemozoin crystals.

While high‐pressure freezing followed by freeze substitution significantly improves the preservation of cellular ultrastructure, it still typically relies on staining with uranyl acetate and lead citrate to enhance membrane contrast. This staining requirement may limit the applicability of HPF‐FS protocols in high‐resolution volume electron microscopy, where consistent and strong contrast is critical throughout large image stacks. In contrast, several room‐temperature chemical fixation protocols have been optimised with enhanced en bloc heavy metal staining steps, enabling excellent membrane visualisation without the need for post‐staining.^72^, ^73^ Recent methodological advances extended this strategy to HPF‐FS workflows, combining cryo‐preservation with improved contrast.^74^, ^75^ These protocols involve rehydration of HPF‐FS samples followed by an aqueous solvent‐based metal staining protocol based on the combination of osmium and thiocarbohydrazide staining. This integrated approach has yielded consistently enhanced membrane contrast across diverse samples, including plant tissues, nematodes, and yeast,^75^ thus expanding the potential of HPF‐FS for high‐contrast 3D imaging in a wide range of biological systems.

Overall, our findings show that the improved preservation of *Plasmodium* spp. cells reveals novel morphological characteristics, potentially providing new insights into parasite structural organisation. The protocol described in this study was validated using two murine models of malaria. However, given the conserved biochemical composition of membranes across *Plasmodium* species, it is likely to be broadly applicable, particularly to *P. falciparum*, the most virulent and deadliest malaria parasite infecting humans. Nevertheless, high‐pressure freezing and freeze‐substitution techniques require specialised equipment that is not widely available in most research labs. It is important to recognise that conventional electron microscopy preparation protocols remain valuable tools for the ultrastructural analysis of the parasite, especially in studies that do not seek fine details on structural organisation.

## Supporting information



Supporting Information
