# Supplementary material for: Optimisation of freeze substitution protocols for the examination of malaria parasite structure by volumetric electron microscopy
Source: J Microsc. 2025 Jul 9;301(2):168–86. doi: 10.1111/jmi.70007 (PMC12884447; doi:10.1111/jmi.70007)
Supplement: Supplementary file 1 — Supporting Information [file JMI-301-168-s001.docx]

Supplementary material:


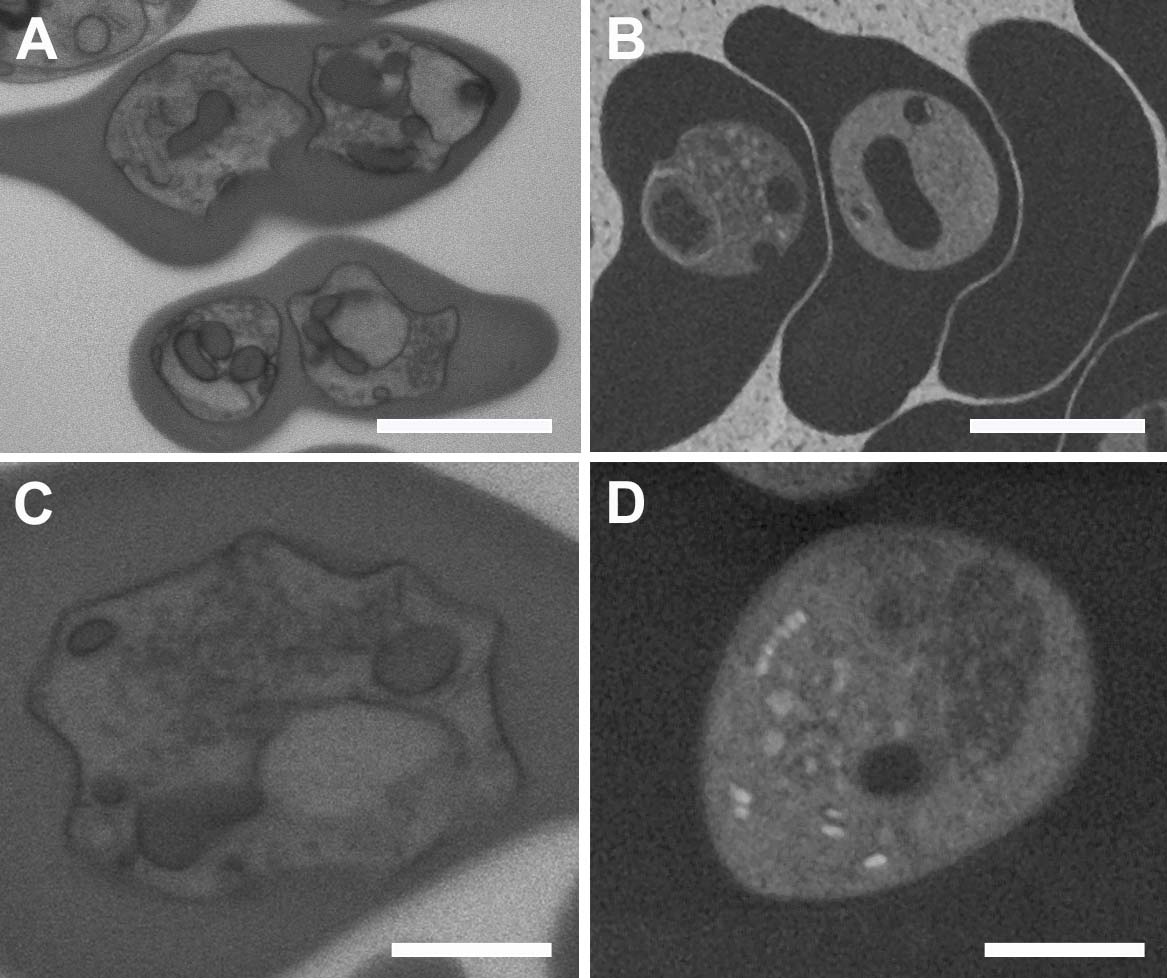


Figure S1. Representative 2D FIB-SEM image slices of CF (A, C) and HPF-FS) samples. Images were acquired at low magnification to provide an overview of cellular morphology. This setting limits the resolution of subcellular structures but allows comparative visualization of general preservation quality between fixation methods. Scale bars: 1 µm (A-B), 500 nm (C-D).


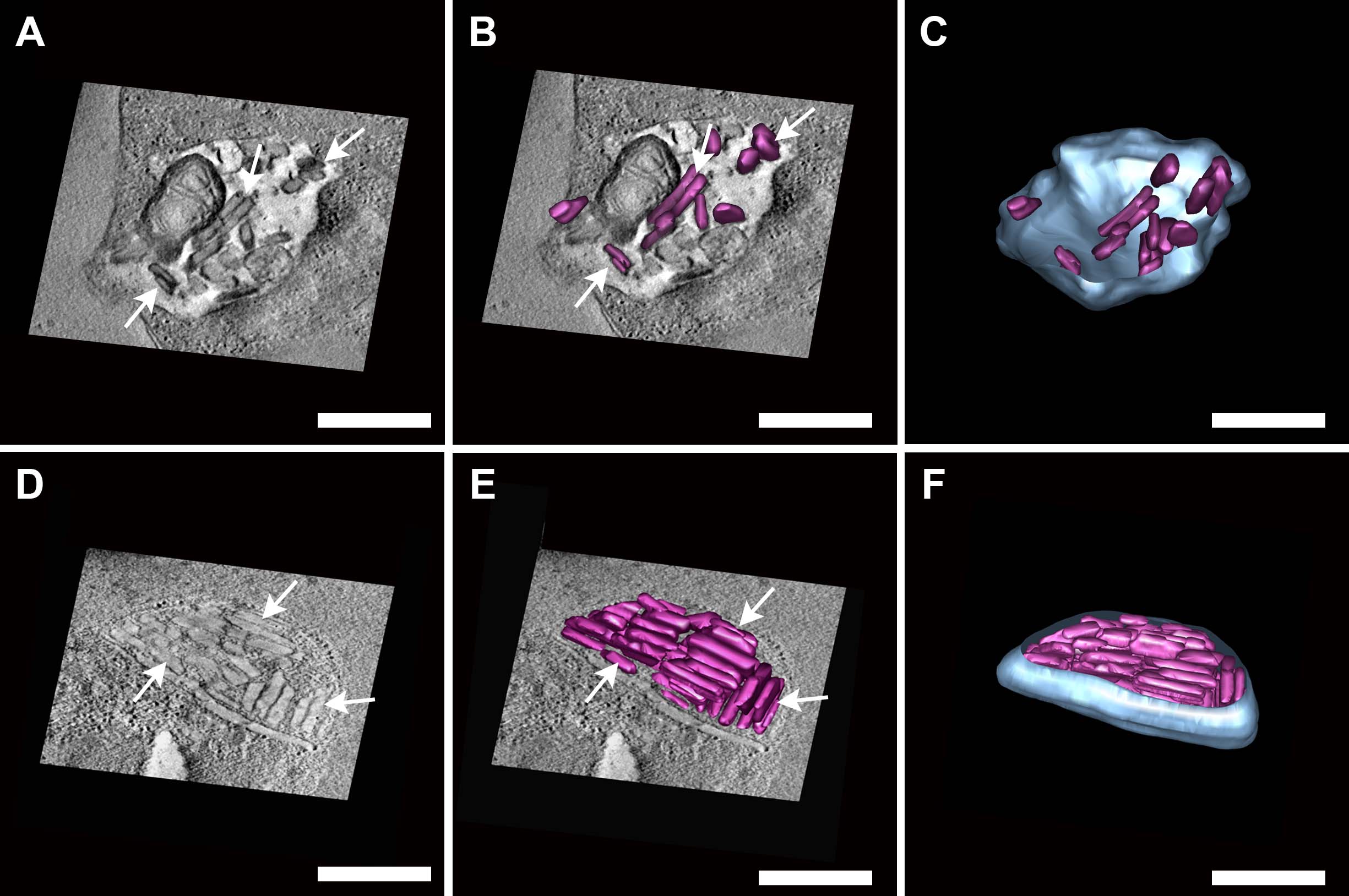


Figure S2. Electron tomography of hemozoin crystal distribution within the food vacuole in chemically fixed (CF) and high-pressure frozen/freeze-substituted (HPF-FS) samples. CF samples exhibit food vacuoles with ruffled membranes (A-C) and few hemozoin crystals on its interior A–B, arrows; C). HPF-FS samples, on the opposite, displayed food vacuoles with a smoother membrane (D–F) and densely packed with hemozoin crystals (D–F, arrows; F). Light blue: food vacuole, purple: hemozoin crystals. Scale bar: 500 nm.
